# Antegrade CT pyelography of right retrocaval ureter causing ureteral stenosis and ureterohydronephrosis in an exotic shorthair cat: A case report

**DOI:** 10.3389/fvets.2022.1013230

**Published:** 2023-01-04

**Authors:** Bowen Zhang, Mengzhu Fu, Di Zhang, Yipeng Jin, Gang Liu

**Affiliations:** ^1^Department of Clinical Veterinary Medicine, College of Veterinary Medicine, China Agricultural University, Beijing, China; ^2^Medical Imaging Department, China Agricultural University Veterinary Teaching Hospital, Beijing, China

**Keywords:** retrocaval ureter, ureterohydronephrosis, ultrasonography, computed tomography, antegrade pyelography

## Abstract

Retrocaval ureter is a rarely reported congenital malformation of the caudal vena cava in veterinary medicine. In this report, a 2-year-old exotic shorthair cat weighing 3.4 kg was presented for depression and loss of appetite. Laboratory findings was unremarkable. Abdominal radiography revealed right renomegaly, and ultrasonography suggested right ureterohydronephrosis. Right retrocaval ureter was recognized by computed tomography. An antegrade pyelography was performed to identify the localization of obstruction and whether obstruction was complete or partial. Complete right ureteral stenosis was confirmed through right antegrade pyelography on computed tomography. The cat underwent right nephroureteroectomy and recovered well after surgery. This is the first report of successful diagnosis and treatment of retrocaval ureter in a cat with significant clinical and imaging signs, using ultrasonographically guided percutaneous antegrade pyelography and multimodal imaging such as radiography, ultrasonography, and computed tomography.

## Introduction

Retrocaval ureter, also named circumcaval ureter or preureteral vena cava, has been described as a rare anomaly of the embryonic venous system in cats (1). It occurs when a persistent right cardinal vein traps part of the ipsilateral ureter dorsal to it. According to previous reports, the prevalence of the disease in feline ranges from 16 to 35.2% (2–4). Such anatomic abnormalities may be incidental without any clinical manifestations. However, in some cases, clinical signs can be found, such as abdominal pain, ureteral obstruction and ureterohydronephrosis (5). There is little literature about this disease in clinical practice, and this study presented the successful diagnosis and treatment of right retrocaval ureter in a cat with significant symptoms, based on radiography, ultrasonography, computed tomography and antegrade pyelography.

## Case report

The affected animal was a two-year-old male exotic shorthair cat weighing 3.4 kg who had been routinely immunized and dewormed. He had no prior medical history and in single-cat households. In recent days, He had been evaluated for a two months' history of vomiting with the frequency of once every half month. He appeared depressed and suffered from a loss of appetite. On physical examination, he experienced mild pain on abdominal palpation. The remainder of the examination was unremarkable.

## Investigations

**Figure 1:**
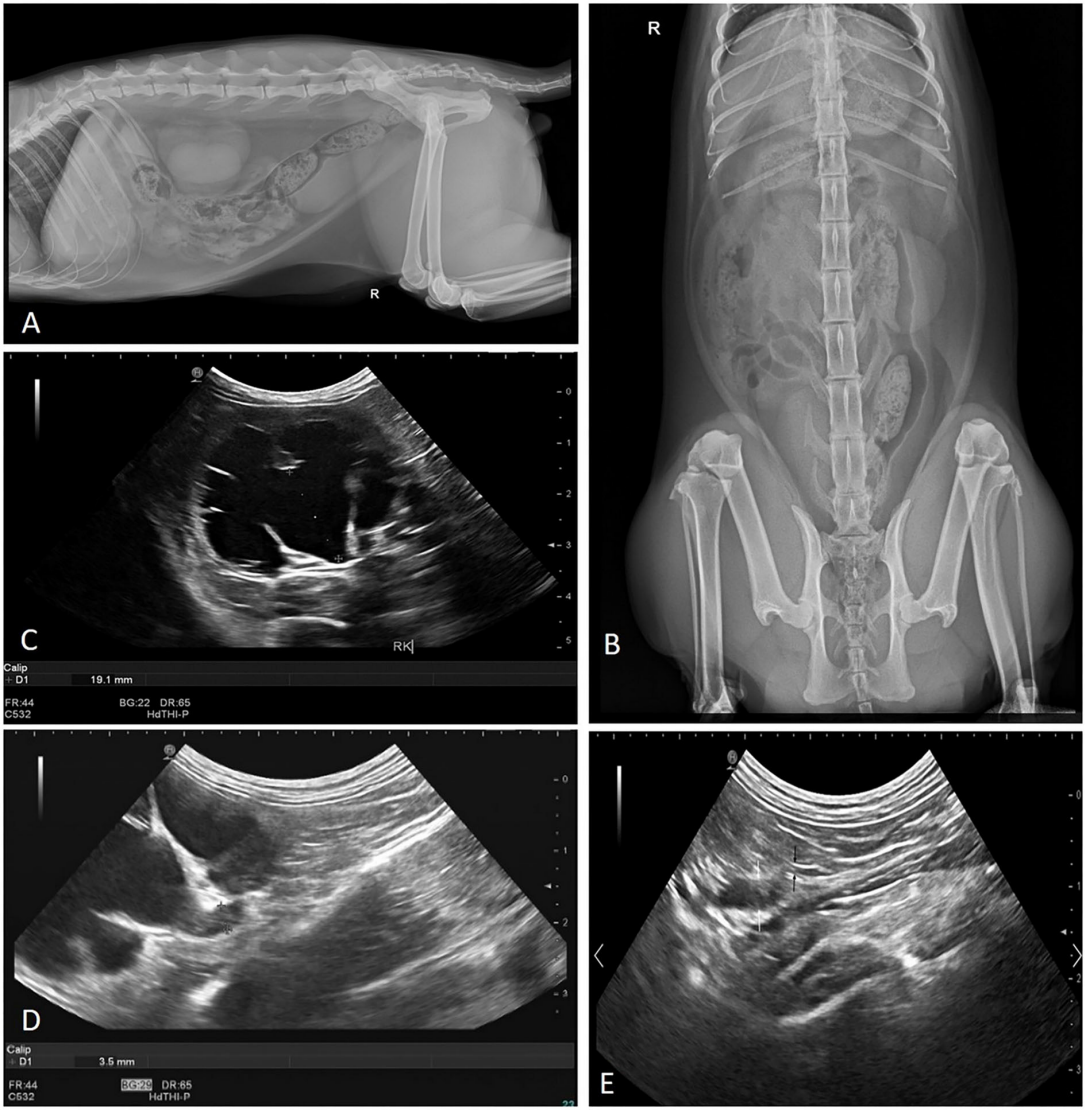
Abdominal radiography and ultrasonography revealed: **(A)** enlarged unilateral kidney in the right lateral view, **(B)** a right renomegaly confirmed in ventrodorsal view, **(C)** transverse image of the right kidney demonstrated severe renal pelvic dilation (calipers), **(D)** dilatation of right proximal abdominal ureter (calipers), and **(E)** right proximal ureter dilatated (white arrow) and tapered abruptly (black arrow).

Complete blood count analysis, blood gas examination and biochemical examination were performed and the results were within the normal range (blood urea nitrogen, 9.0 mmol/L, reference range, 3.5–10; creatinine, 101.2 μmol/L, reference range, 45–135). Urinalysis of a sample collected by cystocentesis revealed urine specific gravity of 1.043, urine pH of 6.3, and no white blood cells or red blood cells. An aliquot of the same urine sample was submitted for microbial culture and a negative result was obtained. The results of the tests for feline leukemia virus and feline immunodeficiency virus were negative. On the abdominal radiography (ROTANODE E7884X, Toshiba Electron Tubes & Devices CO., LTD., Tokyo, Japan), right renomegaly was confirmed (Figures 1A, B). On abdominal ultrasonography (HI VISION Avius L, Hitachi, Tokyo, Japan), the overall contour of the right kidney was smooth (5.58 cm in length) while severe hydronephrosis was observed. Dilatation of the right renal pelvic (1.91 cm at its greatest diameter) and the right proximal ureter (0.35 cm at its greatest diameter) were present (Figure 1C). The dilated proximal ureter extended about 1.45 cm and then narrowed abruptly (Figure 1D). The caudal ureter was indistinct (Figure 1E).

**Figure 2:**
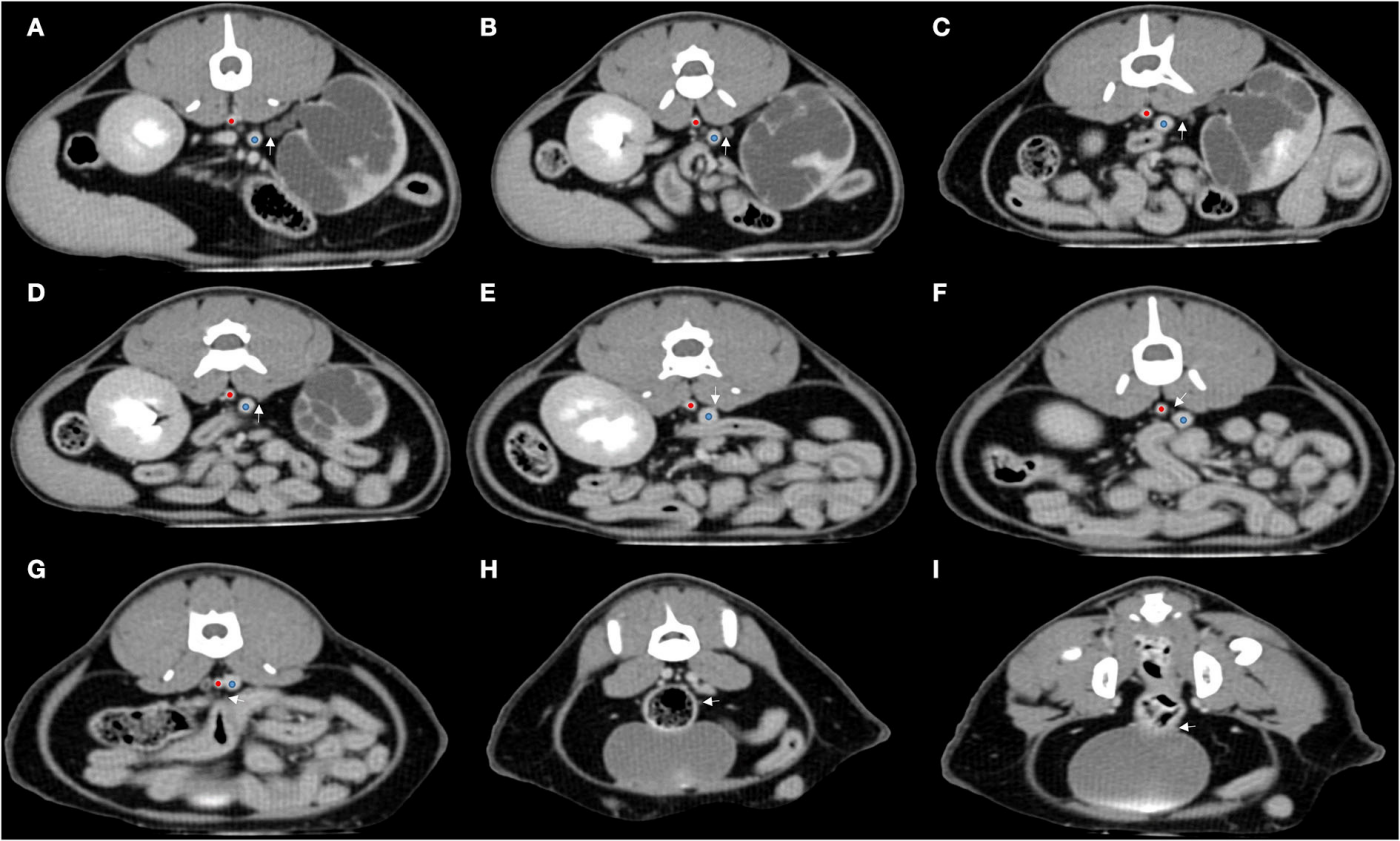
Transverse MPR post-contrast reconstructions from cranial **(A)** to caudal **(I)** of the abdomen using a soft tissue algorithm at 30 s after contrast. It indicated that the right circumcaval ureter ran (white arrow) dorsally to the caudal vena cava. After that, it ran medially and then ventrally toward the gap between the caudal vena cava (blue dot) and abdominal aorta (red dot).

A full body contrast enhanced CT scan (MX 16-slice, Philips, Amsterdam, Holland) with intravenous contrast medium application was performed under general anesthesia. Anesthesia was induced with propofol (5 mg/kg IV) and butorphanol (0.2 mg/kg IV), and maintained with sevoflurane. The CT scans were performed before contrast injection and after contrast for 10 s, 30 s, 110 s, 5 min, 12 min, and 20 min. During each phase of contrast enhancement, the renal cortical CT attenuation number of the normal kidneys was 20–40 HU more than that of the right kidney. The CT scan confirmed that the right renal pelvis irregularly dilated with a maximum diameter of 2.43 cm. The right proximal ureter dilated with a diameter of 0.40 cm, and tapered (0.09 cm in diameter) at the level of fourth to fifth lumbar vertebrae (L4–5). It ran dorsally toward the caudal vena cava at this level and continued ventrally between the aorta and the caudal vena cava at the level of sixth lumbar vertebrae (L6), finally inserted into the urinary bladder (Figure 2). Post-contrast images confirmed that only a tiny amount of contrast medium was observed in the right kidney at 20 min after the injection of contrast medium, which did not drain into the proximal ureter. In contrast, contrast medium started to drain into the left ureter 5 min after the injection.

**Figure 3:**
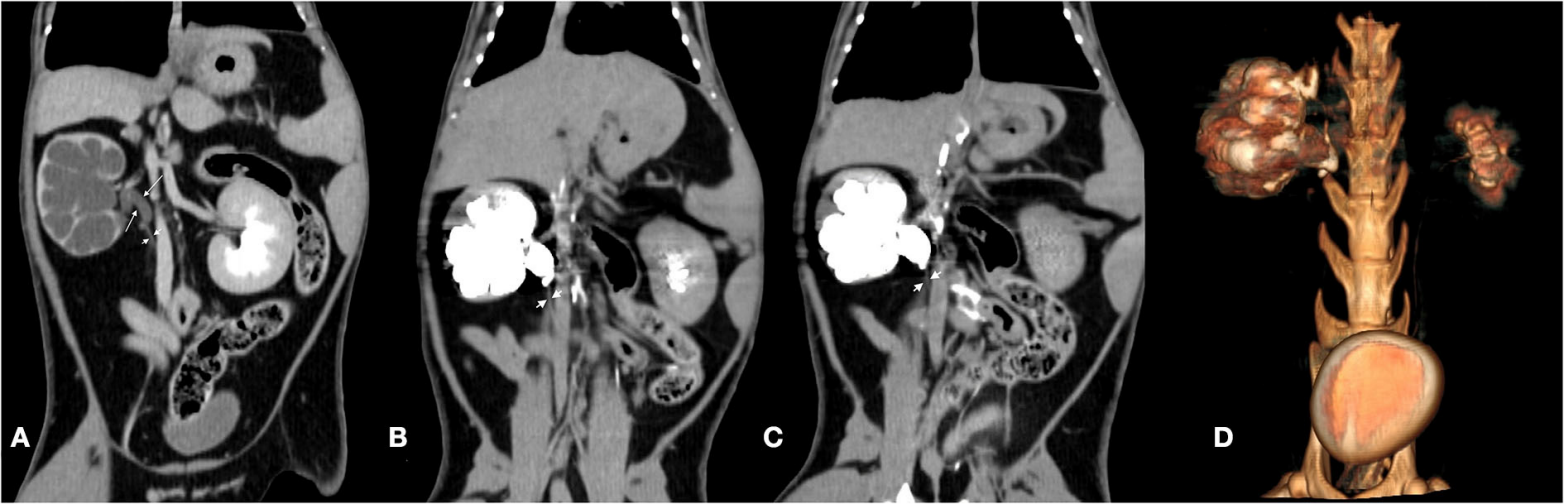
Dorsal oblique MPR post-contrast reconstructions and 3D volume rendering image of the abdomen before and after the antegrade pyelography. **(A)** Before the antegrade pyelography, the dilated right proximal ureter (long arrow) and taper distal ureter (short arrow) was detected. **(B)** 0 min after the puncture. **(C)** 5 min after the puncture. Distal ureter was not filled with contrast material and tapered (short arrow). **(D)** 3D volume rendering image showed the shapes of renal pelvis after the antegrade pyelography.

To clarify the diagnosis, ultrasonographically guided antegrade pyelography was performed on the CT table. The kidney was stabilized by firm pressure applied to the transducer for imaging in the longitudinal plane. A 22-gauge needle was introduced into the renal cortex, perpendicular to the capsule, and advanced into the renal pelvis during ultrasonographic guidance. Depending on the degree of dilatation of the renal pelvis, urine (10 ml) was aspirated from the renal pelvis. An equal volume of ionic, iodinated contrast material was introduced into the renal pelvis in multiple small boluses as the operator of the syringe subjectively assessed resistance to injection. After administration of contrast material, the needle was withdrawn. The CT scans of the kidney were performed at 0 min and 5 min after the puncture, respectively (Figure 3). It was observed that the contrast column interrupted at the level of L4–5 where the ureter tapered, and no contrast medium was observed in the distal ureter. The urine sample (5 ml) collected by antegrade pyelography was submitted for urinalysis and microbial culture and a negative result was obtained.

In summary, the radiography and ultrasound examination suggested the right ureterohydronephrosis may lead to the cat's depression and loss of appetite. However, mechanical obstruction caused by calculus or masses was not detected by the ultrasound scan in this case. Further diagnosis was made by the CT examination which confirmed a right retrocaval ureter. Notably, antegrade pyelography revealed the localization of a complete ureteral stenosis and indicated the reason of right hydronephrosis. The distal segment of the right ureter did not enhance after contrast, which confirmed the absence of excretory function of the right kidney. Therefore, a right nephroureterectomy was considered as a prior treatment option.

## Treatment

**Figure 4:**
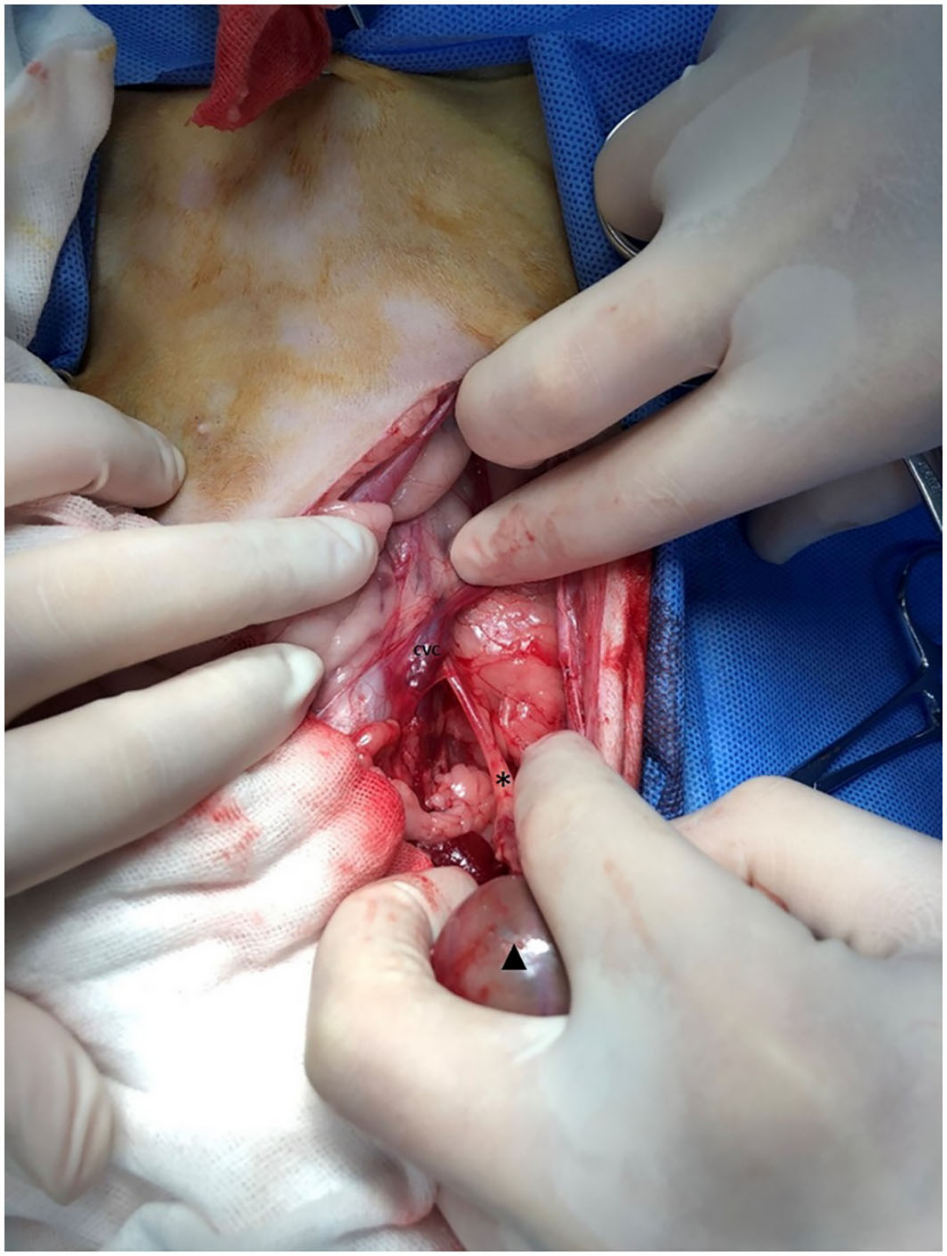
Intra-operative image of the right kidney (triangle) and hydroureter (asterisk). The ureter rans dorsally to the caudal vena cava (CVC).

A right nephroureteroectomy was performed (Figure 4). The affected kidney was dissected from its retroperitoneal sublumbar attachments. The right kidney was enlarged with unsmooth contour, and the cortex became thin. The right ureter ran dorsally toward the caudal vena cava at the level of L4–5. All branches of the renal artery were identified and double ligated with 4/0 absorbable suture close to the abdominal aorta to ensure that all branches had been ligated. The renal vein was ligated similarly. After transaction of the renal vessels, the dilated right ureter was dissected and ligated close to the bladder using 4/0 absorbable suture. Thereafter, subcutaneous tissues were closed with a simple continuous pattern of absorbable suture material, and the preputialis muscle fibers was reapposed and the skin was closed. Cefazolin (25 mg/kg IV q12 h) were administered for the prophylactic management of postoperative infections.

## Outcome and follow-up

Postoperatively, the cat was routinely rehydrated. Butorphanol (0.2 mg/kg SQ q8h) was injected to relieve postoperative pain, and omeprazole sodium (1 mg/kg IV q24h) was administered for vomiting symptoms. After surgery, the cat appeared slightly depressed, and there was no prominent vomiting with a moderate appetite and drinking. Urine output was normal. Two weeks later, the patient remained without clinical signs. Six months later, the owner described him as in good spirits. Two years later, the patient came to the hospital for medical checkups. The image of bladder, the left kidney and the left ureter was normal on ultrasonography. Urinalysis of a sample collected by cystocentesis revealed urine specific gravity of 1.045, urine pH of 6.5, without white blood cells or red blood cells.

## Discussion

In human medicine, the prevalence of retrocaval ureter was 0.13% (6). In small animal medicine, retrocaval ureter has been reported in dogs (7, 8). However, this abnormality was more common in cats, with one study showing a prevalence of the disease between 16 and 35.2% in two feline studies (3, 4). In addition, the anomaly usually occurred in cats in conjunction with other congenital anomalies, such as portosystemic shunt, double right renal vein, duplication of the caudal vena cava, and left-sided caudal vena cava (1, 5). Cryptorchidism had been reported to be associated with this rare condition in the literature in human medicine (9). In the present case, the affected cat had bilateral subcutaneous cryptorchidism. The relationship between retrocaval ureter and cryptorchidism in cat had not been previously described which warranted future research.

The retrocaval ureter is considered as a result of a congenital malformation of the vena cava. The embryonic CVC consists of three embryonic venous systems, caudal or posterior cardinal, subcardinal and supracardinal veins, which develop, regress and anastomose during fetal development (10). In humans, the inferior vena cava is usually thought to develop from the right subcardinal vein located dorsal to the ureter while the posterior cardinal vein regressed. If the posterior cardinal vein does not regress, allowing the ventral segment of the perimetanephric ring to form the inferior vena cava, the right ureter will lay beneath the cava, thus leading to the appearance of the retrocaval ureter (11).

There are two main types of retrocaval ureter: low loop (Type I), in which the dilated upper ureter descends from the renal pelvis, then curves medially and upwards (usually at the level of third to fourth lumbar vertebrae), generating a reversed J, S or hook appearance on the retrograde pyelogram; high loop (Type II), in which the renal pelvis and the upper part of the ureter are nearly horizontal, causing the retrocaval ureter to be on the same level as the renal pelvis, with a sickle-shaped appearance on the intravenous pyelogram (10, 11). The second type is usually non-obstructive due to the passage of a thicker renal artery between the inferior vena cava and the spine, resulting in a wider gap (12). In contrast, the first type is more common and often leads to obstructive sighs, such as significant hydronephrosis and proximal ureteral dilatation (10). In cases of the retrocaval ureter in cats, the most common location for the ureter to cross the caudal vena cava was at L4–5 (2). In accordance with previous publication, the site where the ureter tapered and traveled dorsal to the caudal vena cava was exactly L4–5 in this case.

When the retrocaval ureter presented in cats, many cases did not show clinical signs, and this congenital variant was usually an incidental finding (13). If the ureter was obstructed, it may show corresponding clinical findings, such as ureteral dilatation, dilated renal pelvis or even azotemia (5). In the present case, the affected cat had right-sided hydronephrosis due to a right ureteral obstruction (proximal ureter dilated, ~0.35 cm in diameter, with no significant distal dilatation) and a successive dilatation of the right renal pelvis (~1.91 cm in diameter). We implied that the reason why the cat only appeared depression rather than azotemia was the left renal compensation.

In human medicine, a common technique used to diagnose the retrocaval ureter is an intravenous pyelogram (10). Ultrasound is usually unable to diagnose the disease, because ultrasound scanning is too operator-dependent for such delicate structures as the ureter, and accuracy of diagnosis may vary among institutions (11). Moreover, it is usually more likely to detect secondary manifestations such as hydronephrosis and ureteral dilatation rather than the relative position of the ureter to the caudal vena cava, therefore the retrocaval ureter is not easily diagnosed. In this case, the right hydronephrosis and proximal ureteral dilatation were identified by ultrasound, as well as a sudden tapering and stenosis of the affected ureter at a point of proximal ureter while the cause of the stenosis remained undetermined. Further CT scan was performed in this case. Contrast-enhanced CT imaging was the preferred method for diagnosing retrocaval ureters (14). CT allows more direct visualization of the vasculature and its association with the upper urinary tract compared to radiography and ultrasound. Generally, the excretory (or pyelographic or urographic) phase generally occurs 5–20 min after contrast medium administration (1). In this phase, while the intensity of the nephrogram declines, contrast medium excretion results in opacification of the renal pelvis and ureters and, later, the urinary bladder. In this case, the CT scans were performed at 5, 12, and 20 min after contrast, and we observed that only a tiny amount of contrast medium was visualized in the right kidney of the affected cat, which was not excreted into the proximal ureter.

To better visualize the contrast-enhanced right ureter, we considered a right antegrade pyelography. Because the right kidney lost its excretory function, the right ureter could not enhance after contrast enhancement. Therefore, the CT image with contrast injection detected only the course of the right ureter rather than the localization of an obstruction. Antegrade pyelography can provide excellent filling of the renal collecting system, regardless of renal function of the animal. In our case, the cat was rescanned at 0 and 5 min after the puncture, respectively. We observed that the contrast column traveled to the dilated proximal ureter and stopped at the level of L4–5, where the ureter crossed dorsally to the caudal vena cava (generating a hook appearance), further suggesting the presence of a retrocaval ureter with complete ureteral obstruction in the affected cat (15). When ureteral obstruction is suspected, ultrasonographically guided percutaneous antegrade pyelography can provide excellent visualization of the affected renal pelvis and ureter, allowing for localization of an obstruction and identifying whether obstruction is complete or partial (16).

According to the literature, in cases of ureteral obstruction, the glomerular filtration rate (GFR) may decrease by up to 54% after 14 days of ureteral stenosis (2). GFR values will not improve after the cause of the obstruction is addressed because experimental evidence has suggested that decreased GFR associated with early ureteral obstruction progresses rapidly to irreversible renal injury after 6 days (2). Therefore, it indicated that if chronic obstruction of the ureter occurs, GFR may not improve well even after recovery. In cats with retrocaval ureter, the preferred resolution was either subcutaneous ureteral bypass (SUB) or nephroureterectomy, depending on the assessment of the function of the affected kidney. The subcutaneous ureteral bypass device reduces the long-term complications of ureteral stents, and has been described to be associated with lower mortality than both traditional surgery and ureteral stents in cats (17). The most common complication in the present study of SUB was device occlusion (3, 4, 17). The outcome of subcutaneous ureteral bypass device placement was dictated largely by severity of the underlying renal disease. In our case, it was diagnosed that the right kidney had lost almost all excretory function, and the right ureter was completely obstructed. Considering the complications, postoperative care of SUB and economic constraints, the owner chose the right nephroureterectomy after discussion with the vets and the cat recovered well after surgery.

In conclusion, the retrocaval ureter is a congenital anatomic anomaly in cats that may or may not be associated with clinical signs, which indicate a possible ureteral obstruction. Conventional diagnostic modalities (radiography or ultrasonography) are often difficult to detect this anomaly, and a contrast-enhanced CT scan is highly recommended for a definitive diagnosis. This report highlighted the employment of antegrade pyelography to have a better visualization of obstruction of ureter, regardless of renal function. Surgical interventions, such as subcutaneous ureteral bypass or nephroureterectomy, are usually recommended if clinical symptoms are present.

## Data availability statement

The original contributions presented in the study are included in the article/supplementary material, further inquiries can be directed to the corresponding authors.

## Ethics statement

Ethical review and approval was not required for the animal study because the animal was a clinical patient at the Teaching Veterinary Hospital and all documentations occurred within normal treatment or care for the patient. Written informed consent was obtained from the owners for the participation of their animals in this study.

## Author contributions

BZ: conception, design and drafting the article. GL and YJ: critical revision of the manuscript and management the clinical case. DZ and MF: editing and reviewing draft. All authors contributed to the article, revising article for reasonable content, and final approval of the completed article.
